# Characterizing Glioblastoma Heterogeneity via Single-Cell Receptor Quantification

**DOI:** 10.3389/fbioe.2018.00092

**Published:** 2018-07-11

**Authors:** Si Chen, Thien Le, Brendan A. C. Harley, P. I. Imoukhuede

**Affiliations:** ^1^Department of Bioengineering, University of Illinois at Urbana–Champaign, Champaign, IL, United States; ^2^Department of Mathematics and Department of Computer Science, University of Illinois at Urbana–Champaign, Champaign, IL, United States; ^3^Department of Chemical and Biomolecular Engineering, University of Illinois at Urbana–Champaign, Urbana, IL, United States; ^4^Carl R. Woese Institute for Genomic Biology, University of Illinois at Urbana Champaign, Urbana, IL, United States; ^5^Department of Biomedical Engineering, Washington University, St. Louis, MO, United States

**Keywords:** single-cell, glioblastoma, RTK, heterogeneity, VEGFR, EGFR, IGFR, stem cell

## Abstract

Dysregulation of tyrosine kinase receptor (RTK) signaling pathways play important roles in glioblastoma (GBM). However, therapies targeting these signaling pathways have not been successful, partially because of drug resistance. Increasing evidence suggests that tumor heterogeneity, more specifically, GBM-associated stem and endothelial cell heterogeneity, may contribute to drug resistance. In this perspective article, we introduce a high-throughput, quantitative approach to profile plasma membrane RTKs on single cells. First, we review the roles of RTKs in cancer. Then, we discuss the sources of cell heterogeneity in GBM, providing context to the key cells directing resistance to drugs. Finally, we present our provisionally patented qFlow cytometry approach, and report results of a “proof of concept” patient-derived xenograft GBM study.

## Introduction

GBMs are the most frequent and lethal malignant primary adult brain tumor (Yadav et al., 2018), which presents a critical need to develop new therapeutics. Addressing the dysregulation of RTK signaling pathways offers promise in overcoming GBM lethality (Hawkins-Daarud et al., 2013; Cloughesy et al., 2014; Smith et al., 2016; Massey et al., 2018). RTK dysfunction has been observed in GBM, where these pathways are correlated with tumor cell proliferation (Johnson et al., 2012; Furnari et al., 2015), angiogenesis (Plate et al., 1994; Kuczynski et al., 2011), tumor invasiveness (Giannini et al., 2005; Sangar et al., 2014), and resistance to therapy (Murat et al., 2008; Lu and Bergers, 2013; Popescu et al., 2015). Moreover, these pathways are popular targets for small-molecule inhibitors (Rich and Bigner, 2004; Candolfi et al., 2011). Unfortunately, the clinical benefit of these targeted therapies is limited by drug resistance (De Witt Hamer, 2010; Szopa et al., 2017).

Increasing evidence suggests that drug resistance may be attributed to tumor heterogeneity (variations within an individual tumor) (Saunders et al., 2012; Furnari et al., 2015; Qazi et al., 2017). For example, a landmark study identified tumor subpopulations resistant to therapy prior to treatment by sequencing 4,645 single cells from 19 melanoma patients. This thorough analysis was enabled by single-cell technology, and may have been overlooked with ensemble sequencing (Tirosh et al., 2016). Additionally, a single-cell analysis of patient-derived xenografts (PDXs) of GBM39 also found higher heterogeneity in resistant tumors than in responsive tumors (Shi et al., 2012). In line with these single-cell measurements, we previously discovered, measured, and statistically described heterogeneity in breast cancer xenografts by quantifying vascular endothelial growth factor plasma membrane receptor (VEGFR) concentrations at the single-cell level (Imoukhuede and Popel, 2014). When we combined this quantitative analysis with computational modeling, we arrived at the prediction that tumors having “high” concentrations of plasma membrane VEGFR1 could be resistant to anti-VEGF drugs (angiogenesis inhibitors) (Weddell and Imoukhuede, 2014). Clinical work supports this prediction for colorectal cancer (Weickhardt et al., 2015), and application of this quantification and prediction should offer a new paradigm for biomarker discovery in cancer medicine.

To address the need for quantitative, single-cell analysis of GBM heterogeneity, we apply our optimized and provisionally patented VEGFR quantitative flow (qFlow) cytometry approach to GBM (Imoukhuede and Popel, 2011, 2014; Imoukhuede et al., 2013; Weddell and Imoukhuede, 2014; Chen et al., 2015, 2017; Imoukhuede and Chen, 2018). We describe expanded measurement to several RTKs critical to tumor development. To provide further context we, briefly, review the roles of RTKs in cancer and present connections between RTK heterogeneity and drug resistance. We then present our approach, qFlow cytometry, and report promising findings of a “proof of concept” PDX GBM study.

## Roles of RTKs in cancer

RTKs are widely expressed transmembrane proteins (Cadena and Gill, 1992; Ferrara et al., 2003). Upon ligand binding, they are activated via canonical (Mac Gabhann and Popel, 2007; Sarabipour and Hristova, 2016) and non-canonical (Steinkamp et al., 2014; Chen et al., 2015; Pennock et al., 2016; Mamer et al., 2017) ligand-induced dimerization and tyrosine phosphorylation mechanisms. Importantly, unligated receptors can dimerize (Ruch et al., 2007; Chung et al., 2010; Low-Nam et al., 2011; Lin et al., 2012; Comps-Agrar et al., 2015; King et al., 2016; Sarabipour and Hristova, 2016; Sarabipour et al., 2016) and signal (Wu et al., 2010; Sarabipour et al., 2016; Kazlauskas, 2017), although ligand binding stabilizes the dimeric receptor structure. These receptor-initiated signaling events regulate cell survival, proliferation, differentiation, and motility (Hubbard and Miller, 2007; Volinsky and Kholodenko, 2013).

VEGFRs are upregulated in many cancers (Ferrara, 2002; Kut et al., 2007; Mac Gabhann and Popel, 2008). Signals through endothelial VEGFRs and the neuropilin (NRP) co-receptors (Imoukhuede and Popel, 2011, 2012, 2014; Imoukhuede et al., 2013; Gelfand et al., 2014) induce the sprouting angiogenic hallmarks of cell proliferation and cell migration (Simons et al., 2016). These sprouting angiogenesis hallmarks also sustain tumor growth and enable tumor metastasis (Hanahan and Weinberg, 2011; Shibuya, 2014). VEGF and other pro-angiogenic factors, may also regulate vascular growth and regression in tumors that co-opt pre-existing blood vessels (Holash et al., 1999; Jayson et al., 2016; Kuczynski et al., 2016).

In addition to these canonical pathways, cross-family signaling may also affect tumor vascularization. In this paradigm, ligands from one growth factor family bind to and signal through receptor(s) of another family. For instance, we have shown VEGF-mediated downregulation of PDGFRs (Chen et al., 2015), and discovered that both VEGF–PDGFR binding and PDGF–VEGFR binding is high affinity (Mamer et al., 2017). Other cross-family studies have identified VEGF–PDGFR binding and signaling (Ball et al., 2007; Pennock et al., 2016) and VEGFR–PDGFR dimerization in tumor associated pericytes (Greenberg et al., 2008). Altogether, these canonical and cross-family RTK mechanisms suggest several possible receptor activation landscapes that can contribute to tumor growth and drug resistance.

## Gbm-associated cell heterogeneity: stem and endothelial

An accepted origin of tumor heterogeneity involves clonal evolution; an reiterative process of genetic mutation, clonal selection, and expansion, which drives the growth of single cancer cells into heterogeneous tumor masses (Greaves and Maley, 2012; Greaves, 2015; McGranahan and Swanton, 2017). In addition to cancer cells, other cell types within the tumor may also differentiate or transition as tumor develops. Some such cells include: tumor-associated fibroblasts, macrophages/monocytes, endothelial cells (ECs), and stem cells (Saunders et al., 2012). Here, we describe glioblastoma stem cells (GSCs) and ECs, which we focus on in our pilot study.

GSCs are an important tumor cell component, because despite their small number (~0.5–10%) (Pallini et al., 2011), GSCs are more resistant to radiotherapy and chemotherapy than other cancer cells (Schonberg et al., 2014; Seymour et al., 2015). Furthermore, their resistance can amplify tumor heterogeneity, because they have self-renewing and tumor-initiating capabilities (Lathia et al., 2015). GSCs are often identified by CD133 (Mak et al., 2011), which is associated with poor prognosis in a number of tumor types. There is controversy surrounding the usage of CD133 as a GSC marker (Golebiewska et al., 2013; Seymour et al., 2015; Bradshaw et al., 2016). Early studies showed a subpopulation of GBM cells expressing CD133 were able to form tumors (Singh et al., 2004) and further studies showed subpopulations of CD133^−^ cells were also able to form tumors *in vivo* (Beier et al., 2007). While these studies do not negate the possible role of CD133 in identifying GSCs, they do highlight the importance of heterogeneity and the need for additional markers. Therefore, establishing a “barcode” of RTK plasma membrane concentrations on GSCs may help to identify novel markers, aiding in the isolation and understanding of these stem cells.

ECs, the primary structural unit of the vasculature, are an important contributor to GBM development. Unlike normal vessels, tumor vasculature is leaky, tortuous, and dilated (Jain, 2005; Aird, 2009). In addition to typical tumor vascular pathological features, brain tumor vasculature exhibits the loss of the important blood-brain-barrier feature of tight EC-EC junctions when tumor size grows beyond 1–2 mm in diameter (Jain et al., 2007). The close interaction between tumors and tumor vessels, and the observation of extensive EC heterogeneity supports the need for profiling tumor-associated ECs.

## A paradigm shift in single-cell technologies: from gene-centric to proteomics

Studies characterizing GBM heterogeneity primarily focus on genetic and transcriptomic profiling (Verhaak et al., 2010; Snuderl et al., 2011; Dunn et al., 2012; Szerlip et al., 2012; Brennan et al., 2013; Patel et al., 2014; Ellis et al., 2015), which does not always correlate with functional changes (Simonson and Schnitzer, 2007; Feng et al., 2009; Taniguchi et al., 2010). Moreover, multiple studies show discordance between sequence data and protein expression in GBM, particularly with regards to epidermal growth factor receptor (EGFR) (Brennan et al., 2009) and PDGFR (Hermanson et al., 1992) gene vs. protein expression. Because proteins are the effectors of signaling toward functional response (Grecco et al., 2011; Imoukhuede et al., 2013; Chen et al., 2017), there is a need for increased protein-based, functional measurements.

qFlow cytometry offers a powerful tool for protein-based, single-cell measurements. It applies fluorescent calibration to traditional flow cytometry, converting signal to absolute protein concentrations (Lyer et al., 1997; Lee-Montiel and Imoukhuede, 2013; Chen et al., 2017). Absolute protein quantification allows detection of variations in proteins across published studies, tissues, replicates, and instrument settings (Wheeless et al., 1989; Rocha-Martins et al., 2012; Baumgartner et al., 2013; Nguyen et al., 2013; Vigelsø et al., 2015). Moreover, qFlow cytometry advances systems biology, providing the quantitative data needed for computational studies (Chen et al., 2014; Weddell and Imoukhuede, 2018). For example, using qFlow cytometry coupled with systems biology, we predicted that anti-VEGF efficacy depends on tumor endothelial VEGFR1 plasma membrane concentrations (Weddell and Imoukhuede, 2014). Furthermore, a receptor-internalization computational model recently predicted that small increases in plasma membrane RTK concentrations (< 1,000 receptors/cell) may double nuclear-based RTK signaling (Weddell and Imoukhuede, 2017), which further implicates RTK concentrations as a determinant of signal transduction. These predictions were only possible with the accurate experimental data offered by qFlow cytometry.

## A new approach for examining GBM heterogeneity

**Figure 1:**
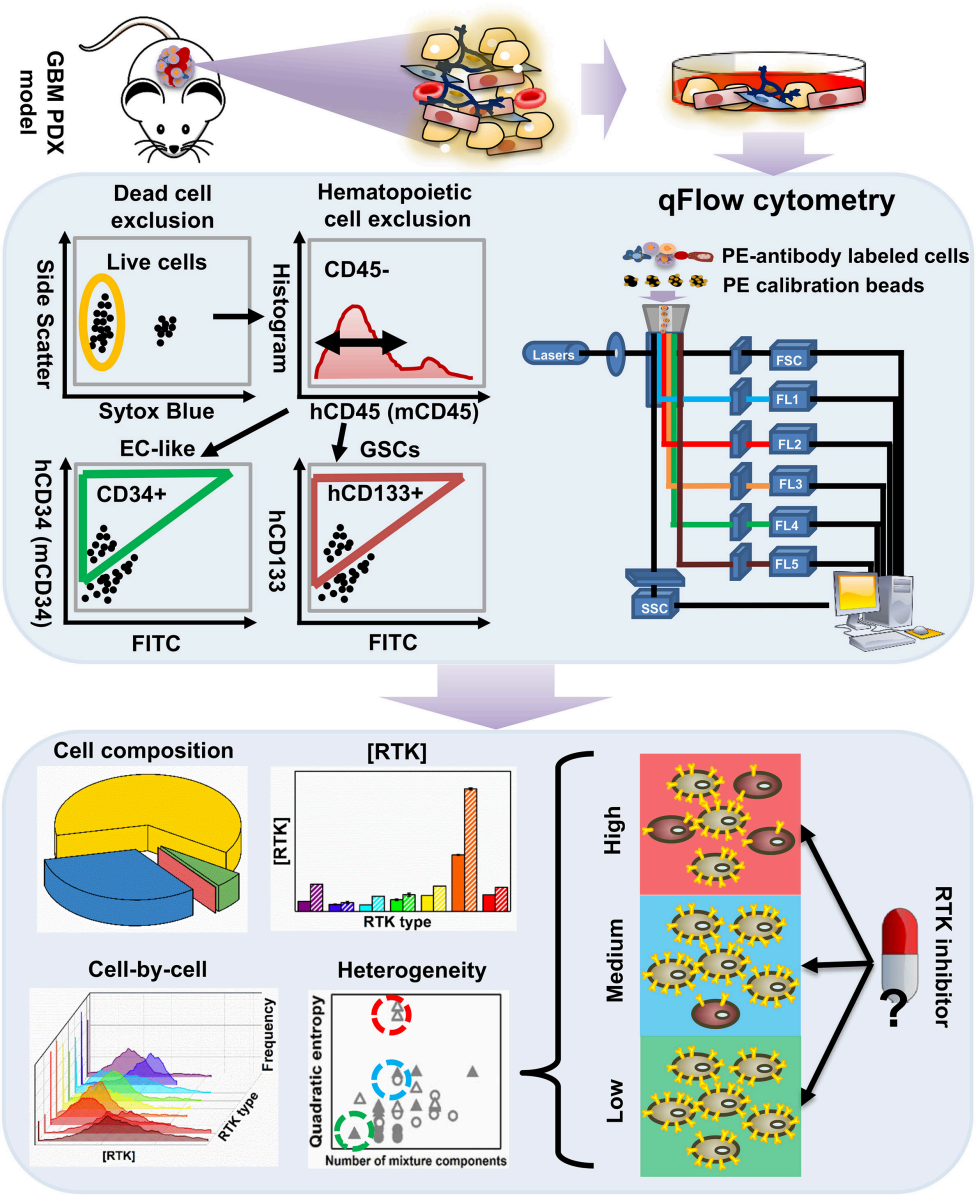
An overview of the workflow for characterizing tumor heterogeneity in GBM39 PDX samples. The GBM39 PDX is established with tumor tissue from patients at Mayo Clinic, Rochester, MN. Following dissociation, multi-channel flow cytometer is used to characterize PDX cells. Briefly, dead cells are excluded using a live/dead cell stain, and hematopoietic cells are excluded using the CD45 antigen, then the endothelial marker CD34 and CD133 can be used to identify EC-like cells and GSCs respectively from the CD45^−^ pool. Percentage of GSCs, EC-like cells and other PDX cells within all live cells can be exported from the flow cytometer. Cells are also stained with phycoerythrin (PE)-conjugated antibodies targeting one of the 9 plasma membrane RTKs: established GBM biomarkers, EGFR and IGFR, and those within the angiogenic signaling networks, VEGFRs, PDGFRs, NRP1, and Tie2. qFlow cytometry is performed as described previously, and ensemble averaged plasma membrane RTK concentrations and cell-by-cell RTK distributions can be obtained (Imoukhuede and Popel, 2011; Chen et al., 2015, 2017). We use two parameters to quantify RTK heterogeneity across EC-like and non EC-like cells: number of mixture components and Quadratic entropy of the cell-by-cell RTK distribution. Bayesian Information Criterion (BIC)-guided Gaussian mixture modeling is used to select the best number of mixture components existed in a larger cell population based on their RTK concentration. Alternatively, Quadratic entropy sums the weighted differences of the means between two bins from 500 equally distributed bins from each cell-by-cell distribution. We envision that characterizing RTK heterogeneity may help understand why RTK inhibitors have not been efficient in treating GBMs.

We performed a “proof of concept” qFlow cytometry study on a PDX, GBM39 (Figure 1). GBM39 is known for EGFR^vIII^ and low invasiveness, *in vivo* (Johnson et al., 2012; Wei et al., 2016). The xenograft was established with tumor tissue from patients undergoing surgical treatment at Mayo Clinic, Rochester, MN. Multiple studies characterize these PDX models and report maintenance of patient morphologic and molecular characteristics including EGFR amplification as well as tumor invasiveness (Giannini et al., 2005; Sarkaria et al., 2007).

**Figure 2:**
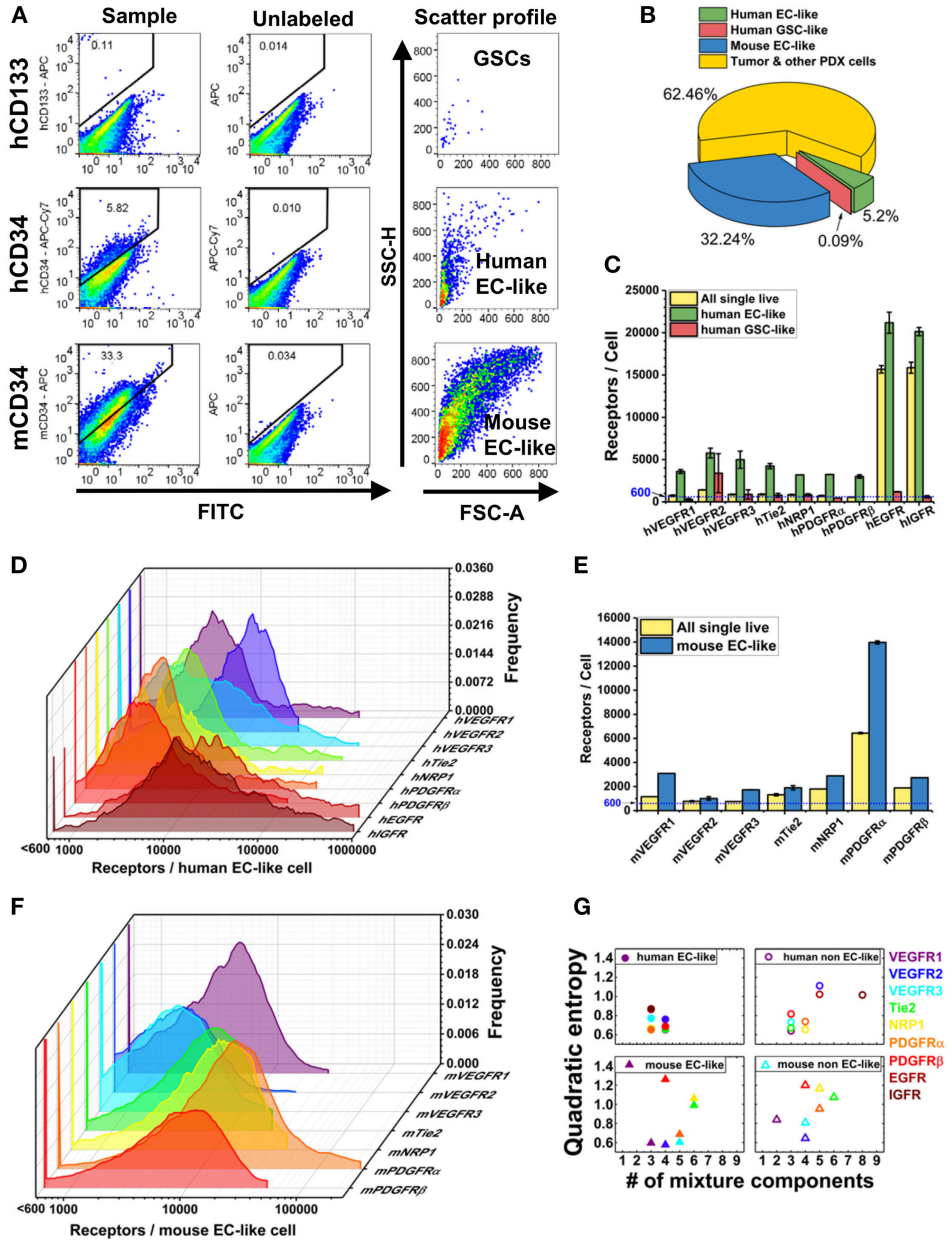
Characterization of plasma membrane RTK concentrations and tumor heterogeneity in GBM39 PDX sample using a LSR Fortessa flow cytometer (BD). We obtain fluorescence signal from 2 to 3 sample tubes for each RTK with 10,000–35,000 live single cells per sample tube. BD FACSDIVA software was used for data acquisition, and FlowJo (TreeStar) software was used for data analysis. **(A)** Representative flow cytometry plots for gating GSCs (hCD45-hCD133+), human EC-like cells (hCD45-hCD34+), and mouse EC-like cells (mCD45-mCD34+) from live cell population. **(B)** Percentage of GSCs, human EC-like, mouse EC-like, and tumor & other PDX cells in the GBM39 PDX sample. **(C)** Ensemble-averaged concentrations and **(D)** cell-by-cell distributions of plasma membrane VEGFRs, Tie2, NRP1, PDGFRs, EGFR, and IGFR on human EC-like cells. **(E)** Ensemble-averaged concentrations and **(F)** cell-by-cell distributions of plasma membrane VEGFRs, Tie2, NRP1, and PDGFRs on mouse EC-like cells. **(G)** Heterogeneity analysis of RTKs in EC-like and non EC-like cell populations. Number of mixture components estimates how many cell subpopulations there are having different plasma membrane RTK concentrations. Quadratic entropy represents the diversity of RTK concentrations within EC-like and non EC-like populations.

Following dissociation, PDX cells were stained with Sytox Blue (a live/dead cell stain), CD45 (Patenaude et al., 2010), CD34 (Soares et al., 2007; Moghadam et al., 2015), and CD133 (Singh et al., 2004; Calabrese et al., 2007; Molina et al., 2014; Naujokat, 2014; Soeda et al., 2015) fluorophore-conjugated antibodies that target EC-like cells and GSCs, respectively (Figure 1). This labeling scheme excludes both dead cells and hematopoietic cells and enables identification of human tumor EC-like cells (hCD34^+^), mouse tumor EC-like cells (mCD34^+^), and GSCs (hCD133^+^) from the live CD45^−^ pool (Figure 2A). To obtain reliable data, we obtained fluorescence signals from 2 to 3 samples/RTK with 10,000–35,000 live single cells collected per sample. As expected, the bulk GBM39 PDX sample was primarily non-EC, non-GSC cells (62.46%). In addition, we found 6-fold higher mouse tumor EC-like cells than human tumor EC-like cells (Figure 2B). This quantification aligned with prior studies of GBM xenograft showing ~7.1% EC population (CD45^−^CD31^+^CD34^+^). Consistent with our quantification of GSCs, a primary human study of 37 patients reported a range of 0.5–10% (Pallini et al., 2011), when identifying GSCs using the CD133 marker.

We labeled and screened 9 plasma membrane RTKs on these cells, which included two established GBM biomarkers, EGFR and insulin-like growth factor receptor (IGFR) (Sangar et al., 2014), and angiogenic signaling biomarkers: VEGFRs, PDGFRs, NRP1, and Tie2 (Carmeliet and Jain, 2000, 2011; Ferrara, 2002; Ferrara and Kerbel, 2005; Dudley, 2012). Using qFlow cytometry and statistical models, we quantitatively characterized GBM39 PDX via four patented metrics (Figure 1): cell composition, ensemble RTK concentration, cell-by-cell analysis with Gaussian mixture modeling, and heterogeneity analysis (Imoukhuede and Chen, 2018).

Percentage of gated cell populations were exported using FlowJo software (TreeStar). Ensemble RTK concentrations and cell-by-cell analysis were performed as previously described (Chen et al., 2015, 2017). We then applied Gaussian mixture modeling to identify log-normal sub-populations within each distribution, described by its mean, standard deviation, and density. We reduced the chance of overfitting the subpopulations by using Bayesian Information Criterion (BIC) (Raftery, 1995; Huedo-Medina et al., 2006). A detailed description of heterogeneity quantification is provided in section Quantification of Cell-RTK Heterogeneity.

### Human tumor EC-like cells have high EGFR and IGFR on plasma membrane

EGFR and IGFR are expressed on tumor cells and contribute to tumor progression. Interestingly, the human tumor EC-like population had high plasma membrane EGFR and IGFR concentrations (~21,000/cell and ~20,000/cell, respectively) (Figure 2C), consistent with qualitative findings of higher EGFR on breast carcinoma-derived ECs compared to normal ECs (Amin et al., 2006). Our results of high EGFR on human tumor EC-like cells from GBM39 is also consistent with results of clinical GBM samples (Soda et al., 2011).

The mixture modeling revealed that 8% of human tumor EC-like subpopulations had a ~12-fold higher membrane localization of EGFRs than average. We found a similar pattern for IGFRs in human tumor EC-like subpopulations. Together, the ensemble-averaged data and the mixture modeling indicated significant plasma membrane localization of EGFR and IGFR on human tumor EC-like cells. High concentrations of EGFR and IGFR suggest an opportunity for targeted inhibition, which could be a mechanism for disrupting tumor vessels on GBMs with a similar profile.

### Mouse tumor EC-like cells have similar plasma membrane VEGFR concentrations as healthy mouse ECs from skeletal muscle

VEGFRs are key regulators of tumor angiogenesis, so their quantification can offer insight into the tumor vasculature. Furthermore, as biomarkers of vasculature, these receptors have been proposed as diagnostic biomarkers of anti-angiogenic drug efficacy (Lambrechts et al., 2013; Wehland et al., 2013) with computational (Weddell and Imoukhuede, 2014) and clinical (Weickhardt et al., 2015) support to their use. We found that VEGFR1 and VEGFR2 had similar concentrations and ratios on mouse tumor EC-like cells (~3,100 VEGFR1/cell and ~1,000 VEGFR2/cell) as on healthy ECs obtained from mouse skeletal muscle (Imoukhuede and Popel, 2012) (Figure 2E). This finding of a low VEGFR2:VEGFR1 ratio aligns with a previous study on breast cancer xenografts (Imoukhuede and Popel, 2014); however, the receptor abundance we report here is much lower. These findings of EC-like cells from GBM39 having VEGFRs at levels similar to normal mouse skeletal muscle ECs suggests a need for further quantification of normal brain ECs VEGFR concentrations to establish tissue standards. Similarly, it suggests a need to examine other GBM specimens to identify whether this is a property of co-opted vessels or specific to this GBM strain.

We analyzed the human tumor EC-like population (5.20% of the population, Figure 2B), which should reflect the original tumor vessels from the patient (Figure 2C). We found similar plasma membrane VEGFR1 and VEGFR2 ratios (~3,600 VEGFR1/cell & ~5,800 VEGFR2/cell) as previous reports *in vitro* (Imoukhuede and Popel, 2011; Chen et al., 2015). However, these data show that not all tumors have the same concentrations or ratios of plasma membrane VEGFRs on their endothelium. Importantly, tumor EC-like cells display much greater heterogeneity than normal ECs with subpopulations that have high concentrations of VEGFRs. Indeed, cell-by-cell analysis and mixture modeling of human tumor EC-like cells reveals the existence of a high-VEGFR1 subpopulation (~10%) with ~41,000 VEGFR1/cell, while the highest VEGFR2 subpopulation is ~18,500 VEGFR2/cell, comprising ~35% of the total human tumor EC-like population (Figures 2D,G). The difference in VEGFR2:VEGFR1 ratio and receptor concentrations between human and mouse tumor EC-like population shows a significant level of endothelial heterogeneity. Such data may enable correlations between these tumor vessel regulators and anti-angiogenic drug efficacy.

### Plasma membrane PDGFRs localize on tumor EC-like cells

PDGFRs serve important roles in supporting vasculature in tumor microenvironments (Andrae et al., 2008). We observed lower levels of PDGFRs on human tumor EC-like cell membranes than on mouse (Figures 2C,E). The cell-by-cell analysis and mixture modeling suggests that this ensemble average does not capture the subpopulations having high-PDGFR plasma membrane localization: 66 and 16% of mouse tumor EC-like cell membrane had ~23,400 PDGFRα and ~19,800 PDGFRβ, respectively (Figure 2F). This significant heterogeneity may be attributed to the use of the CD34 marker to designate EC-like cells, because it is also found on stem cells/precursors, mast cells, and neurons (Nielsen and McNagny, 2008; Imoukhuede and Popel, 2014; AbuSamra et al., 2017). PDGFRα is also considered an important mesenchymal stem cell marker (Farahani and Xaymardan, 2015). So, the co-labeling of PDGFRα and CD34 suggests these cells may be mesenchymal stem cells (Aguirre et al., 2010).

If these CD34^+^PDGFR^+^ cells are endothelial, then our data correlates with studies finding PDGFRs on tumor ECs (Hermansson et al., 1988; Werner et al., 1990; Plate et al., 1992). PDGFR localization on ECs is controversial, because it is characteristic of mural cells and not of ECs (Heldin et al., 1981; Bowen-Pope and Ross, 1982; Kazlauskas and DiCorleto, 1985; Raines et al., 1991; Battegay et al., 1994; Marx et al., 1994). However, they have been observed on monolayer microvascular ECs, *in vitro* (Bar et al., 1989; Marx et al., 1994) and on angiogenic ECs that formed sprout and tubes *in vitro* (Battegay et al., 1994). If we subscribe to the canonical PDGFR localization understanding, then these tumor vessels induce “non-conventional” PDGFR localization patterns.

### GSCs have little-to-no surface EGFR or IGFR

Multiple studies suggest that a higher degree of GSC “stemness” is associated with *EGFR* amplification (Mazzoleni et al., 2010; Liffers et al., 2015); however, we observed ~13-fold lower EGFRs on GSC plasma membranes compared to the bulk PDX cells (Figure 2C). This trend was also seen with IGFR (Figure 2C). The low membrane EGFR concentrations on GSCs is concerning, given reports that EGFR signaling is necessary for GSC proliferation and tumor-sphere formation (Soeda et al., 2008; Griffero et al., 2009). Yet, this may explain the lower percentage of GSCs in the PDX sample (~0.9%) compared to the expected stem cell fraction (0.5–10%; Pallini et al., 2011). A possible explanation is that serially transplanted tumors can lose their EGFR overexpression, even *in vivo* (Liffers et al., 2015). Clearly, further investigation of both gene expression and protein quantification on other GBM PDX GSCs is necessary to understand their contribution to heterogeneity and drug-resistance.

### Quantification of cell-RTK heterogeneity

To quantify heterogeneity of each cell subpopulation, we used two parameters: number of mixture components and Quadratic Entropy (QE). To quantitatively assess the number of subpopulations within each cell population, we fit each cell-by-cell RTK distribution with mixture models consisting of 1–9 log-normal Gaussian sub-distributions (mixture components); we then applied BIC as the criterion to select the mixture model with the lowest BIC. The number of mixture components is determined by how many log-normal Gaussian sub-distributions are in the mixture model. The number of mixture components, thus, is a measurement of cell heterogeneity. Generally, 1–2 mixture components are considered low heterogeneity (Chen et al., 2017), while more than two components is considered highly heterogeneous (Imoukhuede and Popel, 2014; Weddell and Imoukhuede, 2014).

Alternatively, QE requires equally spaced bins, here we chose 500 bins, from each cell-by-cell distribution (Figures 2D,F). QE then sums the weighted differences of the means between two bins (Rao, 1982; Pavoine and Dolédec, 2005; Zoltán, 2005). Thus, QE is a measurement of the increase in random variation in the cellular response. Because healthy ECs and human fibroblasts *in vitro* have shown QE within 0.2–0.7 (Chen et al., 2015), we describe QE < 0.7 as low heterogeneity and QE > 0.7 as high heterogeneity. QE provides a quantitative measure of the diversity of cellular phenotypes in cancer tissue sections for diagnostic applications (Potts et al., 2012) and drug discovery (Gough et al., 2014). Interestingly, human tumor EC-like cells showed lower QE and number of mixture components when compared to mouse tumor EC-like cells (Figure 2G). We suspect that the likely loss of human tumor-associated cells over time in a PDX model (Chao et al., 2017) may be the reason why human tumor EC-like cells present a more homogenous state than the mouse tumor EC-like cells.

### Clinical implications of GBM heterogeneity

We envision that RTK quantification can identify ideal receptor targets across the bulk tumor specimen and on specific cell populations in the tumor. First, the ideal receptor target would be highly available (Rich and Bigner, 2004; Weis and Cheresh, 2011; Cloughesy et al., 2014): it would have high concentrations on a high percentage of bulk cells or specific cells. Next, the target RTK would exhibit low heterogeneity: it would have low QE in bulk cells or on the specific cell subpopulation (Jain et al., 2009; Heath et al., 2016). An ideal receptor target would also be highly specific to the tumor, which would manifest as higher receptor concentrations in the tumor vs. healthy tissue (Rich and Bigner, 2004).

Based on these guidelines, we offer possible targets on GBM39. If the goal is targeting tumor vessels, then VEGFR2 and PDGFRα are highly targetable: >70% target cells have > 6,000 VEGFR2 or PDGFRα/cell plasma membrane with QE = 0.20 or 0.32, respectively. Furthermore, they are likely targets, because they are more highly expressed in GBM specimens than health tissue (Chen et al., 2015): ~5-fold higher VEGFR2 and ~4-fold higher PDGFRα. Therefore, targeting VEGFR2 and PDGFRα should preferentially target the tumor.

Our work suggests that targeting EGFR and IGFR on tumors like GBM39 may not be effective by itself. Although, they have high concentrations on ~70–90% EC-like and non-EC-like GBM cells, their high GBM heterogeneity (QE = ~1.0) and high concentration on healthy tissue (2–2,000 × 10^3^ EGFR/fibroblast or epithelial cell; 2.5 × 10^4^ IGFR/NIH 3T3 mouse fibroblasts (Sorkin and Duex, 2010; Brennan et al., 2013; Weddell and Imoukhuede, 2017) may lower their targeting specificity, resulting in lower drug efficacy (Wheeler et al., 2010). Better drug delivery to the tumor site will likely improve targeting specificity without disrupting healthy tissue. An alternative strategy is to develop dual-inhibitors targeting both EGFR/IGFR and VEGFR2 to increase their specificity for tumor EC-like cells.

We believe our method can also identify cellular and molecular mechanisms underlying reduced response to drugs. For example, upregulation of alternative signaling pathways has been implicated in anti-VEGF drug resistance (Bergers and Hanahan, 2008; Shojaei and Ferrara, 2008). This mechanism of drug resistance is often accompanied by significant tumor heterogeneity (Snuderl et al., 2011; Szerlip et al., 2012; Lu and Bergers, 2013). Therefore, these alternative pathways may be overlooked in bulk studies if they are only present on small cell subpopulations. From this study, we suggest targeting RTKs that are localized on plasma membrane at high concentrations on small cell populations (< 10%) for combination therapy. For example, VEGFR1 and Tie2 on tumor ECs may become “alternative” RTKs for anti-VEGF treatment, because we found ~10% human tumor EC-like cell subpopulations had 41,000 VEGFR1 and ~8% had 65,700 Tie2 on the plasma membrane. Identifying alternative RTK pathways that contribute to resistance can provide tumor-specific drug targets for combination therapy.

## Future direction in characterizing GBM heterogeneity

Our study of the GBM39 PDX model, arrived at 4 key findings and 2 recommendations: (1) tumor EC-like subpopulations have high concentrations of plasma membrane VEGFR1 and VEGFR2; (2) human vs. mouse tumor EC-like cells have inverted VEGFR2:VEGFR1 ratios; (3) tumor EC-like subpopulations have high plasma membrane EGFR, IGFR, and PDGFR concentrations; and (4) GSCs compose a low percentage of cells in the tumor and have little-to-no EGFRs and IGFRs on their plasma membranes.

Based on findings in this study and our RTK-targeting criteria, VEGFR2 or PDGFRα would be likely drug targets for GBM39. In addition, VEGFR1 and Tie2 are likely drug targets for combination therapy. The next step would be to test these targets in a GBM PDX model.

The results of this “proof of concept” study should be interpreted as such: it offers an approach for continued measurement of tumor samples, broadly, and GBM samples, specifically, with the GBM39 PDX sample as a first example. We present the novel method, qFlow cytometry, and show its application in characterizing GBM heterogeneity. Larger and well-powered samples are warranted to expand the current preliminary results, and to discover ideal drug targets and mechanisms underlying drug resistance.

Future opportunities for expanding this research lies in establishing protein concentration ranges on additional samples and continued development of biomimetic tumor models. Firstly, additional measurements of protein concentration on normal ECs and other cells would provide the baselines needed to compare to tumor. In establishing EC baselines, isolation of a pure EC population may be a challenge. Previous qFlow studies have identified ECs using both the CD34 and CD31 markers (Imoukhuede and Popel, 2012, 2014; Imoukhuede et al., 2013). However, it is important to note, that using multiple markers can bias cell collection: CD34 is a progenitor marker, so its use biases selection from more mature cells. Whereas, CD31 is a mature cell marker that is found on ECs, platelets, natural killer cells, monocytes, macrophages, and among other cells (Liu and Shi, 2012), so its use can lead to sample impurity. Here, we chose to bias toward progenitor-like ECs; however, expanded studies may determine if protein concentrations correlate with marker presentation (e.g., identifying whether progenitor-like cells having higher or lower protein concentrations).

Another opportunity for advancement lies in our quantitative single-cell RTK mapping, moving toward multiplexed measurement of RTKs. Toward multiplexed quantification, Lee-Montiel et al., developed a quantum dot method for receptor labeling and calibration (Lee-Montiel and Imoukhuede, 2013; Lee-Montiel et al., 2015) that can be translated to qFlow cytometry. Another approach could be to adapt receptor quantification to mass cytometry (CyTOF) (Spitzer and Nolan, 2016). Such advancements would provide multi-RTK, multi-cell insight into tumor heterogeneity.

In conclusion, cancer research is experiencing a paradigm shift from ensemble analysis to cell-to-cell variability (Niepel et al., 2009; Hoppe et al., 2014; Dar and Weiss, 2018) because of the increasing evidence correlating drug resistance with tumor heterogeneity. The perspective and work that we present here offers sensitive methods for heterogeneity characterization in tumors that should enable improved treatment. We believe that continued quantification of single-cell receptor heterogeneity is a new frontier that will offer significant clinical impact.

## Author contributions

SC and PI designed the study. BH provided samples and contributed critical insight into GBM and discussion on the studies. SC performed the experiments and quantified RTK plasma membrane concentrations. SC, TL, and PI conducted and discussed data analysis. SC, TL, and PI prepared the manuscript.

### Conflict of interest statement

The authors declare that the research was conducted in the absence of any commercial or financial relationships that could be construed as a potential conflict of interest.
